# Expression levels of caspase‐3 and gasdermin E and their involvement in the occurrence and prognosis of lung cancer

**DOI:** 10.1002/cnr2.1561

**Published:** 2021-09-23

**Authors:** Yuan‐Li Huang, Guang‐Hui Zhang, Qing Zhu, Xia Wu, Li‐Gao Wu

**Affiliations:** ^1^ Department of Pathology First Affiliated Hospital of Bengbu Medical College Bengbu Anhui China

**Keywords:** caspase‐8, immunohistochemistry, inflammatory, pyroptosis, survival rate

## Abstract

**Background:**

Pyroptosis plays a dual role in the development of cancer and malignancy; as such, it may potentially be a new target for cancer treatment. However, the inflammatory response to pyroptosis may have adverse effects on the body. The roles of gasdermin E (GSDME), caspases, and related proteins associated with pyroptosis in cancer remain controversial.

**Aim:**

The goal of this study was to determine whether the expression levels of caspase‐3 and GSDME affect the clinical stage, pathological grade, or survival prognosis of patients with lung cancer.

**Methods:**

We examined the protein levels of GSDME, caspase‐3, caspase‐8, and caspase‐9 in lung tissue samples from 100 patients with lung cancer by using immunohistochemistry.

**Results:**

We found that GSDME, caspase‐3, and caspase‐8 were more highly expressed in tumor tissues than in adjacent normal tissues. Moreover, we found that GSDME could serve as a prognostic factor as there was a positive correlation between its expression level and the postoperative survival rate of patients with lung cancer.

**Conclusions:**

GSDME may be an independent factor affecting the prognosis of patients with lung cancer. However, the role of GSDME and its related proteins in cancer requires further research.

## 1 BACKGROUND

Over the past decade, there have been significant advancements in research on the epidemiology and prevention of lung cancer through our understanding of the underlying genetics and of the role of the immune system in lung cancer control, and breakthroughs in treatment options. Despite these advances, lung cancer remains the leading cause of cancer‐related deaths.^1^ This may be attributable to detection at later stages, as lung cancer is initially asymptomatic. Furthermore, the high mortality rate may be due to limited treatment strategies for patients with advanced‐stage lung cancer.

Worldwide, lung cancer cases and deaths are on the rise; in 2018, GLOBOCAN estimated 2.09 million new cases (11.6% of total cancer cases) and 1.76 million deaths (18.4% of total cancer deaths),^1^, ^2^ as opposed to 1.8 million new cases and 1.6 million deaths from lung cancer reported in 2012.^3^ It the most common cancer as well as the leading cause of cancer‐related death in both men and women. In women alone, it is the third most common cancer type and the second leading cause of cancer‐related deaths.^1^, ^2^, ^4^ Furthermore, lung cancer rates in developing countries are expected to continue to rise over time.

Pyroptosis is a newly discovered programmed cell death mode that has features of both apoptosis and necrosis. Pyroptosis is a pro‐inflammatory cell death form dependent on the caspase family, and is a programmed cell death pattern.^5^ The role of cell pyroptosis in the development of cancer has attracted a large amount of attention. However, our understanding of pyroptosis is still scanty, and the molecular mechanisms behind pyroptosis and its incidence need to be further explored. At present, pyroptosis is thought to occur via a classical pathway and a non‐classical pathway mediated by an “executive protein” known as gasdermin D (GSDMD). This protein relies on two caspase‐mediated pathways, namely, the caspase‐1 and caspase‐4/5/11 pathways.

GSDMD and gasdermin E (GSDME) belong to the gasdermin family of proteins, which share pore‐forming domains.^6^, ^7^ Unlike GSDMD, the cleavage of GSDME does not involve the caspase‐1 or caspase‐4/5/11 pathways; instead, it relies on another member of the caspase family, caspase‐3. The known caspase‐3‐mediated cell death mode is apoptosis, but when GSDME is present, its expression level, if high, modulates this to lead to pyroptosis instead..^5^ In addition, the mechanisms of cell membrane pore formation by other members of the gasdermin family, such as GSDMA, GSDMB, and GSDMC, remain unclear.

In this study, we explored the role of GSDME and demonstrated that under the mediation of caspase‐3, it splits into the GSDME C‐terminal and the GSDME N‐terminal. The GSDME‐N terminals then accumulate on the cell membrane, leading to the formation of transmembrane pores. This effectively destroys the integrity of the cell membrane, leading to cell disintegration, cell death, and secondary inflammatory reactions. It is noteworthy that caspase‐3 cleaves GSDME but not GSDMD. The expression of GSDME is inhibited in most cancer tissues and as such, GSDME may act a tumor suppressor.^7^, ^8^ Moreover, in breast cancer, a decrease in the levels of GSDME is associated with a decrease in survival rate.^8^, ^9^

## 2 MATERIALS AND METHODS

### 2.1 General information

From January 2013 to December 2014, a total of 100 archived paraffin‐embedded lung cancer specimens confirmed by the Department of Pathology of The First Affiliated Hospital of the Bengbu Medical College were collected. None of the patients with lung cancer received radiotherapy or chemotherapy before surgery. The age range of the patients was 45–81 years, with a median age of 65. All cases were followed up until the death of the patient or until January 2020, with the shortest interval at 60 months and the longest interval at 84 months. This research has been approved by the Ethics Committee of (Blinded per Author Guidelines), and follows the ethical guidelines of the Helsinki Declaration.

All the patients enrolled in this study underwent radical lung cancer surgery, and their specimens were confirmed by the Department of Pathology of The First Affiliated Hospital of the Bengbu Medical College were collected. The baseline data of the selected cases were collected retrospectively; follow up was by telephone. The clinicopathological data of the patients are shown in Table 1.

**TABLE 1:** Summary of patient icohort information

Characteristics	No of cases (%)
Age	
>65	79 (71.8)
≤65	21 (19.1)
Gender	
Male	51 (46.4)
Female	49 (44.5)
Pathological grade	
I	25 (22.7)
II	51 (46.4)
III	24 (21.8)
TNM stage	
I	29 (26.4)
II	31 (28.2)
III	40 (36.4)
Lymphatic invasion	
Yes	43 (39.1)
No	57 (51.8)
Vital states	
Alive	38 (34.5)
Dead	62 (56.4)
Expression of gasdermin E	
Low expression	52 (47.3)
High expression	48 (43.6)
Expression of caspase‐3	
Low expression	12 (10.9)
High expression	88 (80.0)
Expression of caspase‐8	
Low expression	26 (23.6)
High expression	74 (67.3)
Expression of caspase‐9	
Low expression	88 (80.0)
High expression	12 (10.9)
Tumor size	
≤5	74 (67.3)
>5	26 (23.6)
Tumor location	
Central type	64 (58.2)
Peripheral type	36 (32.7)
Tumor types	
Squamous cell carcinoma	67 (60.9)
Adenocarcinoma	31 (28.2)
Small cell carcinoma	2 (1.8)

### 2.2 Reagents

Rabbit polyclonal antibodies against human caspase‐3, caspase‐8, caspase‐9, and GSDME were purchased from Proteintech (Rosemont, IL, USA). The specific information about the antibodies is shown in Table 2. The ElivisionTM Plus Kit and DAB color developing kit were purchased from Fuzhou Maixin Biological Company (China).

**TABLE 2:** Antibody information

Antibody	Catalog Number	Source	Company	Dilution
Caspase‐3	19 677‐1‐AP	Rabbit polyclonal	Proteintech,Rosemont, IL, USA	1:400
Caspase‐8	13 423‐1‐AP	Rabbit polyclonal	Proteintech,Rosemont, IL, USA	1:400
Caspase‐9	10 380‐1‐AP	Rabbit polyclonal	Proteintech,Rosemont, IL, USA	1:200
GSDME	13 075‐1‐AP	Rabbit polyclonal	Proteintech,Rosemont, IL, USA	1:400

### 2.3 Experimental method

All lung cancer tissue samples were fixed in 10% neutral formalin solution. They were routinely collected, paraffin‐embedded, and sectioned at 4 μm thickness. After hematoxylin/eosin and immunohistochemical staining, histological observations were conducted under a light microscope (Olympus light microscope). Clinical staging was performed according to the American Joint Committee on Cancer (AJCC) Cancer Staging Manual (eighth Edition). Immunohistochemical staining using the ElivisionTM Plus Kit was performed according to the manufacturer's instructions.

### 2.4 Evaluation of immunoreactivity

The criteria for scoring GSDME were as follows. The intensity was graded according to the following scale: 0, negative; 1, weak; 2, moderate; and 3, strong. The proportion of the positive tumor cells was graded as follows: 0, <5%; 1, 5%–25%; 2, 26%–50%; 3, 51%–75%; 4, >75%. The final score was computed by multiplying these two primary scores. Final scores of 0–6 were defined as “low expression” or (−); final scores of 6–12 were defined as “high expression” or (+).^10^ The same method was used to evaluate the staining signals for caspase family proteins. The immunohistochemical staining results were determined by two pathologists using an independent double‐blind method.

### 2.5 Statistical analysis

SPSS 25.0 (IBM, Chicago, IL, USA) was used for statistical analysis. The Kaplan–Meier method was used for the survival analysis of caspase‐3, caspase‐8, caspase‐9, and GSDME protein expression groups to draw univariate survival curves. The log‐rank test was used for comparisons between groups, and the Cox multivariate regression model was used for the multi‐factor analysis. In lung cancer tissues, the correlation between the expression levels of caspase‐3, caspase‐8, caspase‐9, and GSDME as well as the clinicopathological parameters were analyzed by the χ2 and Spearman rank correlation tests. Effects were considered statistically significant if *p* < .05.

## 3 RESULTS

### 3.1 Expression levels of GSDME, caspase‐3, caspase‐8, and caspase‐9 in lung cancer tissues and their association with clinicopathological parameters

**FIGURE 1:**
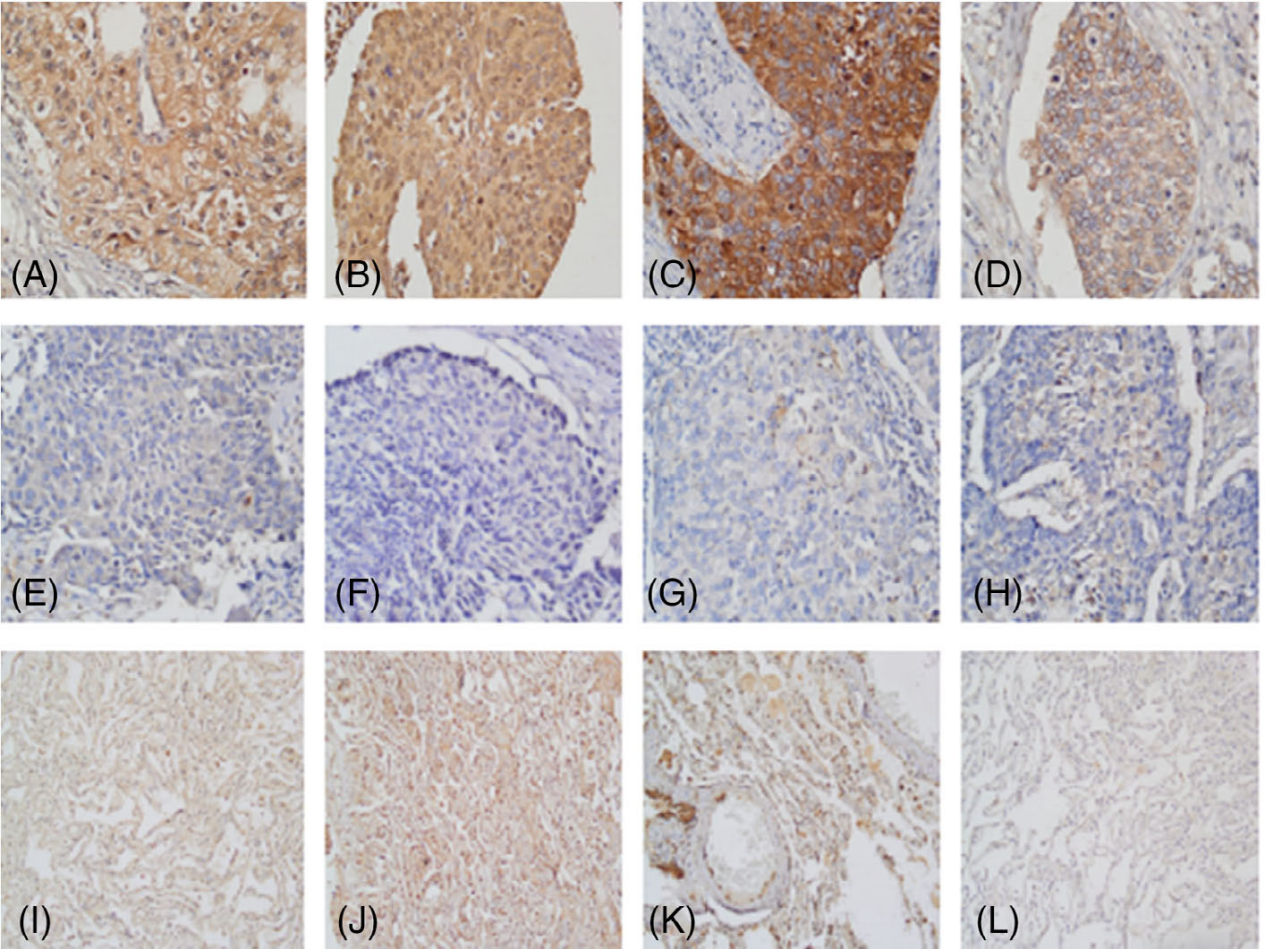
Expression of gasdermin E (GSDME), caspase‐3, caspase‐8, and caspase‐9 in lung cancer. (A) High expression of GSDME in lung cancer tissue. Positive GSDME signal was present in the cytoplasm and cell membrane of cancer cells, which further suggested that GSDME accumulated in the cell membrane, leading to cell membrane pore formation and subsequent disintegration and necrosis. (B) Low expression of GSDME in lung cancer tissue. (C) High expression of caspase‐3 in lung cancer tissue. (D) Low expression of caspase‐3 in lung cancer tissue. (E) High expression of caspase‐8 in lung cancer tissue. (F) Low expression of caspase‐8 in lung cancer tissue. (G) High expression of caspase‐9 in lung cancer tissue. (H) Low expression of caspase‐9 in lung cancer tissue. (Images were acquired at 400× magnification)

Through the analysis of immunohistochemical staining of pathological sections of tissues from 100 patients with lung cancer, we found that the expression level of GSDME in lung cancer tissues was relatively higher than that in the adjacent normal tissues (Figure 1A). GSDME expression levels were found to be higher in the cell membranes of some lung cancer tissues, which is consistent with the possibility that GSDME causes cell disintegration and death by forming holes in the cell membrane.

Table 3 shows the relationship between GSDME expression and the clinical and pathological parameters of the patients. It was observed that patients with high GSDME expression experienced statistically fewer lymph node metastasis and had a significantly higher prognostic survival rate than patients with low GSDME expression. Therefore, GSDME may be an important factor affecting the postoperative survival of patients. In addition, there was a positive correlation between the expression level of GSDME and the stage of lymph node metastasis in patients with advanced tumors. This indicates that GSDME may have an inhibitory effect on tumor growth.

**TABLE 3:** Expression levels of GSDME, caspase‐3, caspase‐8, and caspase‐9 in lung cancer tissues and their associations with clinicopathological parameters

Variable	GSDME			caspase‐3			caspase‐8			caspase‐9		
	Low	High	*p* value	Low	High	*p* value	Low	High	*p* value	Low	High	*p* value
Age												
>65	43	36	0.183	8	71	0.263	22	57	0.414	69	10	0.694
<=65	8	13		4	17		4	17		19	2	
Gender												
Male	27	24	0.692	7	44	0.588	16	35	0.211	44	7	0.588
Female	24	25		5	44		10	39		44	5	
Pathologic grade												
I	10	15	0.135	2	23	0.303	6	19	0.329	24	1	0.341
II	31	20		5	46		11	40		44	7	
III	10	14		5	19		9	15		20	4	
TNM stage												
I	11	18	0.194	4	25	0.88	7	22	0.895	24	5	0.462
II	16	15		3	28		9	22		27	4	
III	24	16		5	35		10	30		37	3	
Lymphatic invasion												
Yes	28	15	.014*	4	39	0.471	11	32	0.934	39	4	0.471
No	23	34		8	49		15	42		49	8	
Vital states												
Alive	12	26	.002*	1	37	.024*	5	33	.022*	31	7	0.122
Dead	39	23		11	51		21	41		57	5	
Tumor size												
<=5	38	36	0.906	9	65	0.933	22	52	0.151	67	7	0.187
>5	13	13		3	23		4	22		21	5	
Tumor location												
Central type	35	29	0.325	7	57	0.663	17	47	0.864	58	6	0.281
Peripheral type	16	20		5	31		9	27		30	6	
Tumor types												
SCC	34	33	0.366	7	60	0.629	18	49	0.694	57	10	0.423
Adenocarcinoma	15	16		5	26		8	23		29	2	
Small cell carcinoma	2	0		0	2		0	2		2	0	
Expression of GSDME												
Low expression	—	—	—	10	41	.017*	17	34	.088	47	4	0.192
High expression	—	—		2	47		9	40		41	8	
Expression of caspase‐3												
Low expression	10	2	.017*	—	—	—	8	4	.001*	11	1	0.677
High expression	41	47		—	—		18	70		77	11	
Expression of caspase‐8												
Low expression	17	9	.088	8	18	.001*	—	—	—	24	2	0.432
High expression	34	40		4	70		—	—		64	10	
Expression of caspase‐9												
Low expression	47	41	0.192	11	77	0.677	24	64	0.432	—	—	—
High expression	4	8		1	11		2	10		—	—	

*Note*:*p <.05 was considered significant.

In this study, the expression of caspase‐9 in lung cancer tissues was lower than expected (Figure 1D). As shown in Table 3, caspase‐9 was weakly expressed in most of the cancer tissues, and the stratified expression levels of the different clinicopathological parameters were not significantly different (*p* > .05). On the other hand, caspase‐3 and caspase‐8 were highly expressed in lung cancer tissues, and they could be considered as possible prognostic factors (Figure 1B, C).

### 3.2 Correlation analysis between GSDME, caspase‐3, caspase‐8, and caspase‐9 levels

To understand whether the expression levels of GSDME, caspase‐3, caspase‐8, and caspase‐9 correlated with each other and whether there were correlations with certain clinicopathological parameters, we calculated Spearman's rank‐order correlation coefficient. The statistical results are shown in Table 4. There was a significant correlation between high expression of GSDME and postoperative survival status, lymph node metastasis, and caspase‐3 (*p* < .05). There was a correlation between the high expression levels of caspase‐3 and caspase‐8; there was also a significant correlation between the high expression levels of both caspase‐3 and caspase‐8 and the postoperative survival status (*p* < .05). There was, unexpectedly, no correlation between the expression of caspase‐9 and other variables; nor was there any correlation between the expression of caspase‐9 and the survival rate of patients.

**TABLE 4:** Correlations between GSDME, caspase‐3, caspase‐8, and caspase‐9 expression levels, and clinicopathological parameters

Variables	GSDME		caspase‐3		caspase‐8		caspase‐9	
	Spearman correlation	*p* value	Spearman correlation	*p* value	Spearman correlation	*p* value	Spearman correlation	*p* value
Age	0.133	0.187	−0.112	0.268	0.082	0.419	−0.039	0.698
gender	0.04	0.696	.054	0.592	0.125	0.215	−0.054	0.592
TNM stage	−0.178	.076	0.01	0.924	−0.002	0.987	−0.124	0.218
Pathologic grade	−0.016	0.878	−0.137	0.175	−0.106	0.295	0.137	0.173
Lymphatic invasion	−0.245	.014*	0.072	0.476	0.008	0.935	−0.072	0.476
Vital states	0.304	.002*	0.226	.024*	0.229	.022*	0.155	0.124
Tumor size	0.012	0.907	0.008	0.934	0.143	0.154	0.132	0.191
Tumor location	0.098	0.33	−0.044	0.667	0.017	0.866	0.108	0.286
Tumor types	−0.062	0.538	0.093	0.36	0.014	0.886	0.099	0.327
Expression of GSDME	—	—	0.239	.017*	0.171	.09	0.131	0.196
Expression of caspase‐3	0.239	.017*	—	—	0.342	<.001*	0.042	0.681
Expression of caspase‐8	0.171	.09	0.342	<.001*	—	—	0.079	0.437
Expression of caspase‐9	0.131	0.196	0.042	0.681	0.079	0.437	—	—

*Note*:*p <.05 was considered significant.

### 3.3 Relationships between the expression levels of GSDME, caspase‐3, caspase‐8, and caspase‐9 and survival rate

Univariate and multivariate Cox proportional hazard models were used to examine the relationships between GSDME, caspase‐3, caspase‐8, and caspase‐9 expression levels, related clinicopathological parameters, and patient survival rates. The corresponding risk ratios were also considered. The statistical results are shown in Tables 5 and 6. The Cox univariate analysis showed that GSDME expression, caspase‐3 expression, TNM staging, lymph node metastasis, and tumor size were all meaningful variables that affected the survival time of patients after surgery.

**TABLE 5:** Cox univariate analysis table

	Univariate analysis		
Variables	*p* value	Hazard ratio	95% confidence interval
GSDME	.002*	0.435	0.258–0.733
Caspase‐3	.017*	0.447	0.231–0.865
Caspase‐8	.073	0.616	0.363–1.046
Caspase‐9	0.119	0.482	0.193–1.205
Age	0.952	1.019	0.553–1.879
gender	0.273	0.755	0.457–1.248
TNM stage	.001*	1.713	1.235–2.375
Pathologic grade	0.18	0.773	0.531–1.126
Lymphatic invasion	.023*	1.788	1.083–2.951
Tumor size	.003*	2.227	1.313–3.776
Tumor location	0.581	0.862	0.509–1.460
Tumor types	0.125	0.673	0.405–1.117

*Note*:*P <.05 was considered significant.

**TABLE 6:** Cox multivariate analysis table

	Multivariate analysis		
Variables	*p* value	Hazard ratio	95% confidence interval
GSDME	0.026*	0.534	0.308–0.926
Caspase‐3	0.126	0.577	0.285–1.167
TNM stage	0.404	1.237	0.751–2.037
Lymphatic invasion	0.623	1.19	0.595–2.379
Tumor size	0.081	1.736	0.933–3.228

*Note*:*P <.05 was considered significant.

**FIGURE 2:**
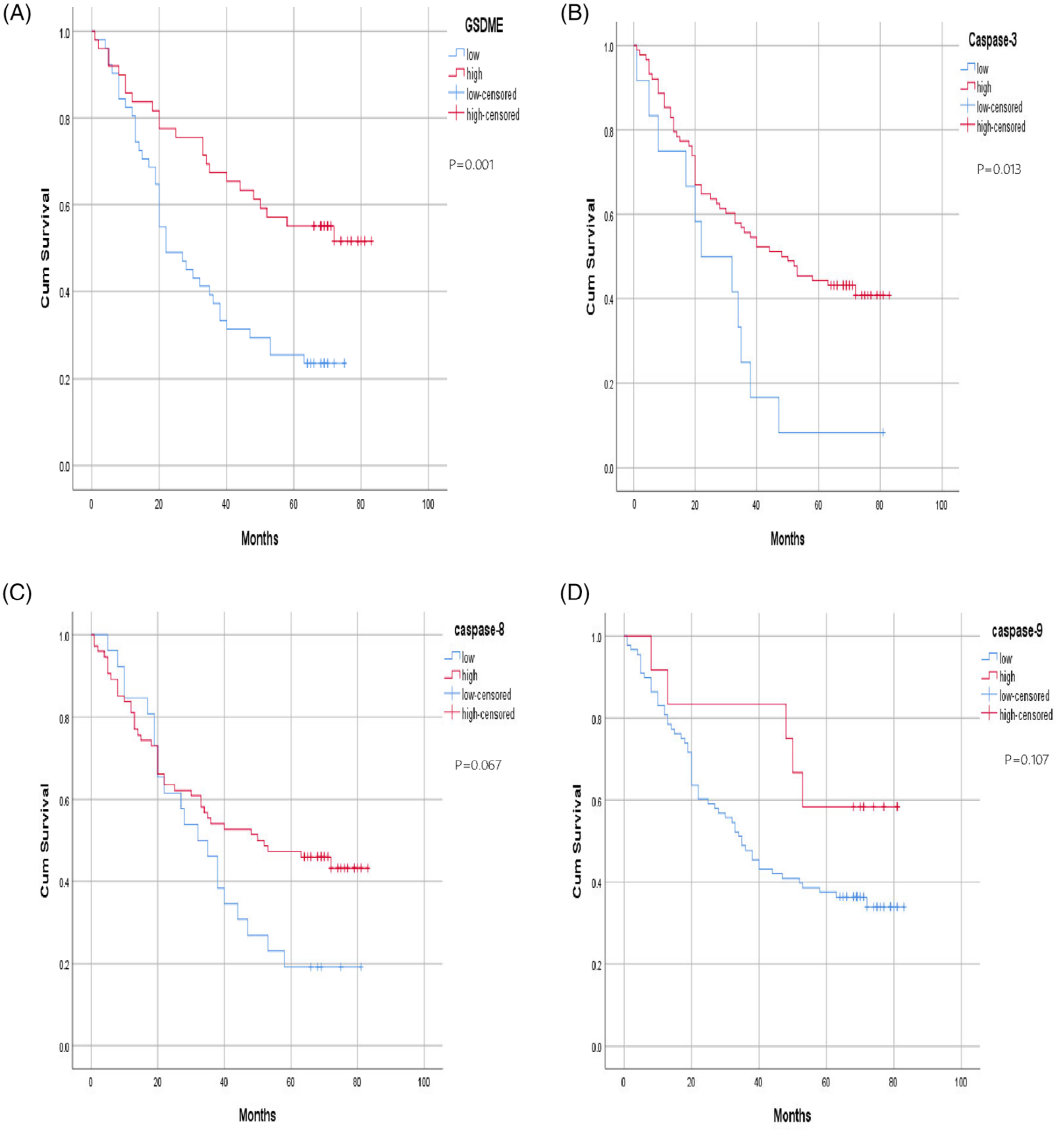
Kaplan–Meier curves of the overall survival of lung cancer patients. The overall survival rates were grouped according to the expression levels of gasdermin E (GSDME) (A), caspase‐3 (B), caspase‐8 (C), caspase‐9 (D), the presence or absence of lymph node metastasis (E), TNM stage (F), tumor composition (G), tumor size (H), tumor location (I), pathological grade of tumor tissue (J), gender (K), and age (L). M. Cox multivariate analysis. The overall survival time of the groups with high expression of GSDME and caspase‐3 (A, B) was significantly higher than that of the groups with low expression levels of these proteins (*p* < .05). The overall survival time of patients without lymph node metastasis and with tumor diameters of ≤5 cm (E, H) was significantly higher than that of patients with lymph node metastasis and tumor diameter > 5 cm (*p* < .05). TNM stage was also an important factor affecting overall survival (F, *p* < .01)

We next conducted the Cox multivariate regression analysis on the above‐mentioned significant influencing factors and their corresponding Cox univariate analyses. We found that not all the significant univariate variables showed significant differences in the multivariate analysis. However, the differences in the expression levels of GSDME remained significant. This confirms that GSDME is not only an influencing factor affecting the survival status, but also an independent prognostic factor of the patients with lung cancer. Thus, GSDME may be used for the clinical treatment and prognostic evaluation of patients with lung cancer. In addition, we plotted the survival curve of each variable and clinicopathological parameters by using the Kaplan Meier survival analysis, as shown in Figure 2.

## 4 DISCUSSION

The incidence and mortality rates of lung cancer have always been high, with surgical resection as the predominant standard treatment. However, the postoperative survival rate of patients is not ideal. The reported 5‐year survival rate for patients with lung cancer was 15.6% in 2011 and 19.4% in 2019.^1^ To achieve a lower mortality rate and a longer survival period, the exploration of lung cancer‐related biomarkers has become a quintessential step in the treatment of lung cancer. In the present study, we explored the possible relationship between cell pyroptosis, the occurrence of lung cancer, and the prognosis of patients with this disease.

Pyroptosis is a form of programmed cell death characterized by cell membrane pore formation, cytoplasmic swelling, membrane rupture, and the release of cytoplasmic contents into the extracellular environment, which amplifies local or systemic inflammation.^11^, ^12^ The pore‐forming proteins of the GSDM family were shown to be involved in the activation of pyroptosis in 2001, and since then, they have been undergoing increased scientific scrutiny.^13^, ^14^ GSDMD was the first protein confirmed to be involved in cell pyroptosis as a substrate of caspases 1, 4, 5, and 11 in humans.^15^, ^16^

In 2017, Shao et al.^6^ found that GSDME, another member of the gasdermin family, also participated in pyroptosis. However, GSDME was activated by caspase‐3,^17^ which is an important factor in the process of apoptosis. It was therefore concluded that cells with high GSDME expression levels are activated by caspase‐3 to redirect caspase‐3‐mediated apoptosis to pyroptosis.^7^, ^13^

GSDME and GSDMD share the same gasdermin N‐terminal structure that gives them the ability to form pores.^7^, ^10^, ^18^ When GSDMD and GSDME are cleaved by caspases, their gasdermin N‐terminal domains translocate and form oligomers in the plasma membrane, thereby leading to the formation of transmembrane pores and the release of cell inclusions.^16^, ^19^, ^20^ The cells then disintegrate and die, causing secondary inflammation.

It has been reported that the expression of GSDME in most cancer tissues is low or even absent.^8^ However, other reports described GSDMD and GSDME expression in a variety of cell types, including epithelial cells (HeLa), kidney cells (HEK293T), melanoma (A375), and lung cells (A549).^16^, ^21^, ^22^ Additionally, in breast cancer, the decrease in GSDME levels is associated with a decrease in the survival rate,^7^, ^8^ indicating that GSDME may be a tumor suppressor. In primary gastric and colorectal cancers, GSDME is inhibited by methylation.^23^, ^24^ GSDME has also been found to be methylated in estrogen receptor‐positive breast cancer and associated with lymph node metastasis.^25^ In esophageal squamous cell carcinoma (ESCC) tissues, the expression level of GSDME has been reported to be higher than in normal esophageal tissues. Therefore, the level of GSDME in biopsy materials can be used as a prognostic indicator of ESCC.^10^

In the present study, we used immunohistochemical staining to analyze the relationship between GSDME expression level and the prognosis of patients with lung cancer. Our results showed that.

high levels of GSDME expression in cancer tissues of patients with lung cancer was associated with a higher survival rate after surgery. In addition, patients in the high GSDME expression group had significantly fewer lymph node metastases. These results are consistent with the above‐mentioned reports suggesting that GSDME may be a tumor suppressor.

In our current study, 110 patients with lung cancer had a high average GSDME expression rate of 49%. From the statistical analysis, we found that patients with high GSDME expression had a longer postoperative survival time and fewer lymph node metastases in advanced tumors. This indicates that low GSDME expression may lead to more aggressive carcinogenic phenotypes. As a tumor suppressor, GSDME may slow down tumor growth and invasion. This is consistent with the findings of a significant increase in cell death in tumors overexpressing GSDME reported by Wang et al.^16^ This may suggest that stimulation of pyroptosis in cancer tissues could be a new direction for cancer treatment. However, the mechanisms behind the inhibition of tumor cell growth by pyroptosis without concomitant destruction of normal body tissues remain to be further studied.

Caspases are a type of cysteine proteases that cleave sites located after aspartic acid residues at specific recognition sites. The activation of these caspases is a biochemical marker of apoptosis.^26^ Apoptosis has been defined as a type of programmed cell death,^27^ which proceeds through two classical signal transduction pathways: the external and internal pathways.^28^ The external pathway is mediated by caspase‐8, whereas the internal pathway is triggered by caspase‐9. Both pathways trigger apoptosis by cleaving the downstream executive protein caspase‐3.^29^

The caspase family is divided into two categories according to the functions of their members in apoptosis (caspase‐3/6/7/8/9) and inflammation (caspase‐1/4/5/12). Caspase‐8 and caspase‐9 are promoters of caspases in the cascade of apoptotic signals; caspase‐3, which is cleaved and activated by caspase‐8 and caspase‐9, is the main executor of caspases.^30^ Caspase‐3 is involved in the regulation of pyrolysis through its function of cleaving GSDME. This means that when GSDME is overexpressed, caspase‐3‐mediated apoptosis is redirected into pyroptosis.^7^, ^13^

However, in this study, we found that only the expression levels of GSDME, caspase‐8, and caspase‐3 were significantly correlated, whereas the expression of caspase‐9 was low in most cancer tissues. We also found that there was no correlation between the expression levels of caspase‐3 and caspase‐8. This was surprising because it is known that caspase‐9 is an upstream mediator of caspase‐3 activation during the mitochondrion‐dependent apoptosis.^31^ This phenomenon indicates that there may be a predominance issue between the actions of caspase‐8 and caspase‐9 upstream of caspase‐3 in some tissues.

In this study, caspase‐8 played a major role with its function upstream of caspase‐3. Furthermore, the high expression levels of caspase‐3 correlated with the high expression level of caspase‐8. Interestingly, caspase‐9 is also reported to be a substrate for caspase‐3 during apoptosis.^31^ However, due to the limitation of conditions, the specific internal mechanisms of the actions of caspase‐8 and caspase‐9 upstream of caspase‐3 have not been explored in this study.

## 5 CONCLUSIONS

Our study found that the expression level of GSDME in lung cancer tissues was higher than in the normal tissues adjacent to cancer tissues. Furthermore, we found that tissues from lung cancer patients with higher GSDME expression had fewer lymph node metastases. Based on the univariate and multivariate analyses, we found that the high expression level of GSDME in lung cancer tissues was associated with longer postoperative survival time, indicating that GSDME may be an independent factor affecting the prognosis of patients with lung cancer. Furthermore, the expression level of GSDME correlated with the expression level of caspase‐3. This study also confirmed that caspase‐8 acts as a promoter, acting upstream of caspase‐3. Based on the univariate analysis, we also found that caspase‐3 is an important factor affecting the postoperative survival time of patients. However, further research is required to elucidate the exact role of GSDME and its related proteins in cancer.

## CONFLICT OF INTEREST

We declare that there are no conflicts of interest on the parts of any of the authors.

### AUTHOR CONTRIBUTIONS

**YL.H:** Data curation (equal); formal analysis (equal); methodology (equal); writing – original draft (lead); writing – review and editing (lead). **GH.Z:** Data curation (equal); formal analysis (equal); project administration (equal). **Q.Z:** Data curation (equal); methodology (equal). **X.W:** Data curation (equal); formal analysis (equal); methodology (equal). **LG.W:** Formal analysis (equal); project administration (lead); supervision (equal); writing – review and editing (supporting).

## Data Availability

Data sharing is not applicable to this article as no new data were created or analyzed in this study.
